# Synergistic modulation of cAMP and cGMP rescues hemin-induced plasma membrane fragmentation

**DOI:** 10.1016/j.jbc.2026.113357

**Published:** 2026-07-24

**Authors:** Zoi Laspa, Anne-Katrin Rohlfing, Melina Fischer, Ferdinand Kollotzek, Tatsiana Castor, Oliver Borst, Pamela Weronika Sowa, Manuel Sigle, Tobias Harm, Meinrad Paul Gawaz

**Affiliations:** 1 Department of Cardiology and Angiology, University Hospital Tübingen, University Tübingen, Tübingen, Germany; 2 DFG Heisenberg Group Cardiovascular Thrombo-Inflammation and Translational Thrombocardiology, University of Tübingen, Tübingen, Germany

**Keywords:** cyclic AMP (cAMP), cyclic GMP (cGMP), cytoskeleton, hemin, plasma membrane, platelet, vasodilator-stimulated phosphoprotein (VASP)

## Abstract

Hemin, which gets released during erythrocyte lysis, induces concentration-dependent platelet activation, aggregation, and thrombus formation as well as plasma membrane destruction and cytoskeleton reorganization. Classical platelet antagonists do not inhibit the plasma membrane destruction induced by high concentrations of hemin. Both cyclic guanosine monophosphate (cGMP) and adenosine 3':5' -cyclic monophosphate (cAMP) are key endogenous inhibitors of platelet activation. Thus, we investigated whether they inhibit hemin-induced plasma membrane destruction, both individually and in combination. In addition to standard platelet assays, we performed cGMP and cAMP ELISAs, immunoblot analysis of vasodilator-stimulated phosphoprotein (VASP) and immunofluorescence staining. We found that pharmacological modulation of these pathways *via* NO donors (DEA/NO), soluble guanylyl cyclase (sGC) stimulators (riociguat) or IP receptor agonists (PGE_1_), and phosphodiesterase (PDE) inhibitors (PDE-5 inhibitor: sildenafil, PDE-3 inhibitor: ibudilast), phosphorylate the downstream vasodilator-stimulated phosphoprotein (VASP) and inhibit hemin-induced platelet activation and degranulation. In particular, synergistically modulation of cGMP and cAMP and the resulted phosphorylation of VASP at Ser239 and Ser157 significantly attenuates platelet aggregation and plasma membrane destruction induced by high concentrations of hemin. Further, the riociguat NO-induced cGMP synthesis was significantly reduced in the presence of hemin. In comparison, the PGE_1_-induced cAMP synthesis was enhanced in the presence of hemin. In conclusion, high concentrations of hemin change the cGMP and cAMP synthesis induced by their associated stimulators, while promoting plasma membrane destruction, which can be significantly inhibited by the simultaneous administration of riociguat DEA/NO and PGE_1_.

Platelets are major players in the regulation of hemostasis and thrombosis. The classic hemostasis is divided into the primary hemostasis that is characterized by platelet adhesion and aggregation at site of vessel injury until a hemostatic platelet plug is formed, whereas during the secondary hemostasis platelets are important for the proteolytic coagulation cascade which leads to plug stabilization by the resulting fibrin mesh. During bleeding events erythrocytes can lyse and release hemoglobin, which is metabolized into free ferrous iron-containing heme and further on oxidized into ferric-iron containing hemin. Hemin is a damage-associated molecular pattern (DAMP) molecule (1, 2). Several studies have demonstrated that hemin induces concentration-dependent platelet activation, aggregation, and platelet-dependent thrombus formation (3, 4, 5). From an evolutionary perspective, locally confined, elevated levels of hemin could be beneficial in initiating primary hemostasis during bleeding events by inducing platelet activation and aggregation. In healthy individuals, free circulating heme and hemin are immediately eliminated by the plasma proteins albumin, haptoglobin, or hemopexin (6). But, in severe hemolytic events these scavengers are not able to neutralize the free hemin, which can result in mayor thrombotic events. It is precisely there that the full proinflammatory and prothrombotic potential of hemin reveals itself.

This prothrombotic effect is initiated by the binding of hemin to the platelet receptors glycoprotein VI (GPVI) and C-type lectin-like receptor 2 (CLEC-2). Platelet activation and aggregation are subsequently induced *via* the classical receptor-dependent activation of the immunoreceptor tyrosine-based activation motif (ITAM) signaling pathway (7, 8). This signaling pathway can be subdued *in vitro* by inhibitors of the ITAM signaling pathway and classical dual antiplatelet therapies. However, these substances are capable of preventing aggregation only when the platelets are stimulated by low concentrations of hemin (3). High hemin concentration, such as those measured during severe hemolytic events, do not respond to classic dual antiplatelet therapies. We have previously postulated that this resistance is caused by a receptor-independent effect of hemin, which predominates at high hemin concentrations. High hemin concentrations were shown to lead to strong changes in the cytoskeleton, morphological changes, lipidome alterations, and plasma membrane disintegration (3, 9). All these changes are triggered by iron-mediated cell death or ferroptosis. The iron-chelator deferoxamine and the inhibition of furin have been shown to inhibit platelet ferroptosis (4, 10). Recently, it was demonstrated that elevated cGMP levels in human platelets contributed as well to the inhibition of hemin-induced platelet aggregation, platelet activation and ferroptotic lipidome changes (5).

The second messenger cyclic GMP and cyclic AMP are two mayor endogenous platelet inhibitors (11, 12). Under physiological conditions, prostacyclin and NO from endothelia cells are activating the adenylyl cyclase (AC) and the soluble guanylyl cyclase (sGC). This leads to elevated cAMP and cGMP levels, which attenuate platelet-dependent thrombosis (13). There are several well-established drugs that can modulate the levels of intracellular messenger cAMP and cGMP and inhibit platelet agonist-dependent calcium mobilization, integrin activation, and degranulation (14, 15, 16). NO-donors, like DEA/NO, or sGC-stimulators (*e*.*g*. riociguat) and sGC-activators (*e*.*g*. cinaciguat) generate cGMP by activating the sGC and further regulate the effectors phosphodiesterase (PDE) and cGMP-dependent protein kinase (PKGIβ), which then phosphorylates substrates like the vasodilator-stimulated phosphoprotein (VASP) (17, 18). PDE-2, a cGMP-stimulated PDE, and PDE-3A cGMP-inhibited PDE, modulate cAMP levels through PDE-mediated crosstalk (19). cAMP levels can also be modulated by the IP3 receptor agonists prostaglandin E1 (PGE_1_). PGE1 binds to the prostacyclin receptor (IP-receptor). The subsequent release of heterotrimeric G-protein (Gs) leads to an enhanced adenylyl cycle activity, which results in increased cAMP levels. cAMP is signaling through PKA and phosphorylates the VASP protein specific at serine 157 (Ser157). In comparison, cGMP-dependent protein kinase phosphorylates the VASP at Ser239 (20, 21). Furthermore, cGMP and cAMP can be regulated through hydrolysis mediated by phosphodiesterases. Three phosphodiesterases (PDE-2A, PDE-3 and PDE-5) are present in platelets. These degradation processes can be blocked by inhibitory substances that target the phosphodiesterase. Sildenafil inhibits PDE-5 and thereby prevents the hydrolysis of cGMP, and ibudilast inhibits PDE-3 and thus blocks the degradation of cAMP (22).

Since cAMP is a potent inhibitory second messenger for platelets and has not previously been investigated in the context of hemin, we analyzed the effects of cAMP-modulating substances, as well as the simultaneous administration of cAMP- and cGMP-modulating compounds, on hemin-induced platelet aggregation, activation, and plasma membrane fragmentation. We used riociguat DEA/NO, a sGC-stimulator, PGE_1_, an IP-receptor agonist and the PDE inhibitors sildenafil (PDE-5 inhibitor) and ibudilast (PDE-3 inhibitor) to modulate cAMP and cGMP levels in isolated human platelets.

This study aimed to investigate the molecular mechanism of the secondary messengers cAMP and cGMP on platelet function, as well as on plasma membrane fragmentation induced by high concentrations of hemin.

## Results

### Modulation of second messengers cAMP and cGMP inhibit hemin-induced platelet aggregation and activation

**Figure 1:**
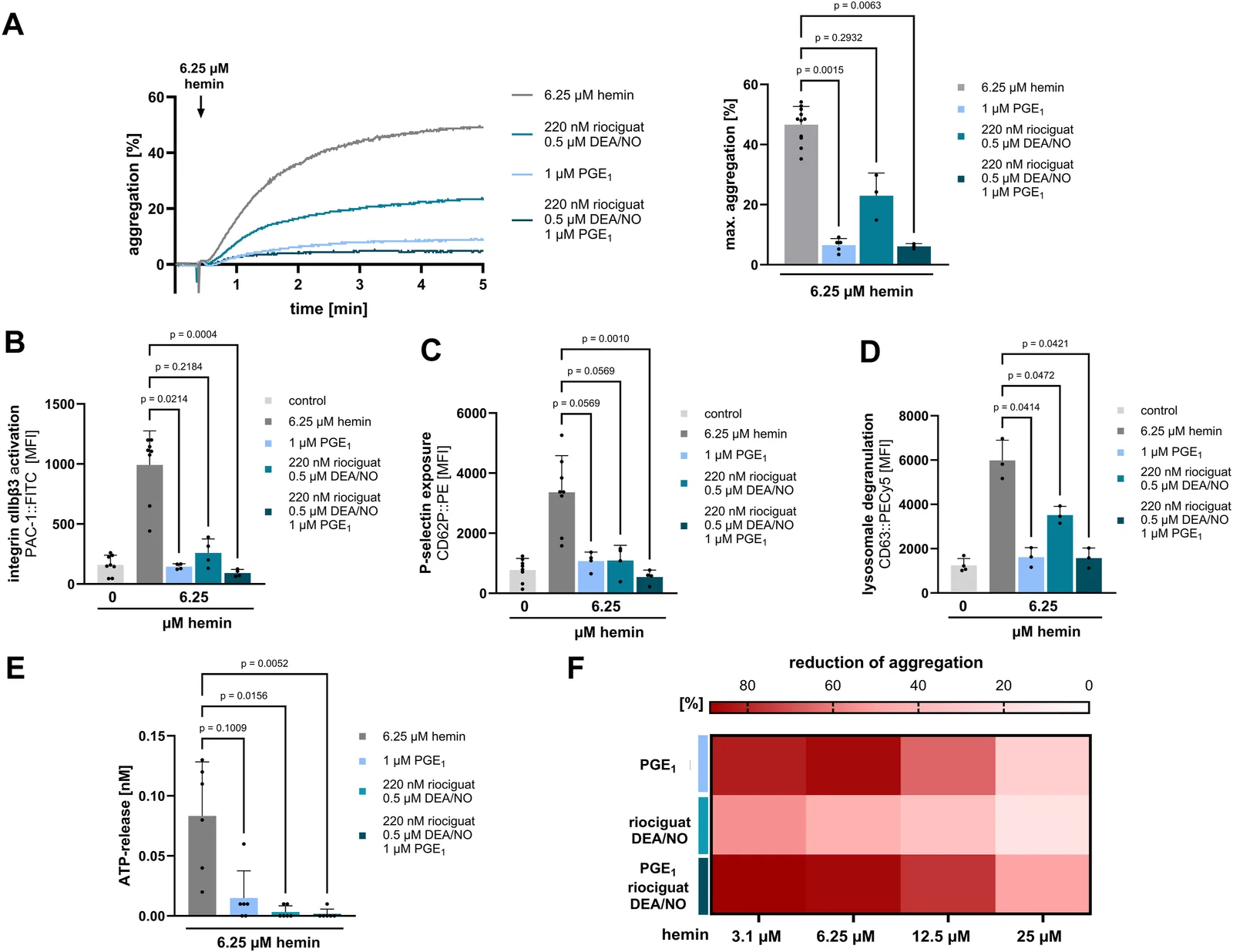
**Modulation of second messengers cAMP and cGMP inhibit hemin-induced platelet aggregation and activation.***A*, light transmission aggregometry measurements. *Left*: Representative traces of 6.25 μm hemin-induced platelet aggregation and if indicated preincubated with 1 μm PGE_1_ (5 min), 220 nm riociguat (5 min) + 0.5 μm DEA/NO (2 min) or the combination (PGE_1_ + riociguat + DEA/NO) at 37 °C. *Right*: Hemin-induced maximal aggregation after 5 min at 37 °C and preincubation with 1 μm PGE_1_ (5 min), 220 nm riociguat (5 min) + 0.5 μm DEA/NO (2 min) or the combination (PGE1 + riociguat + DEA/NO); plotted: Mean ± SD; n ≥ 3; statistics: Kruskal-Wallis test; Dunn`s multiple comparison. *B–D*, flow cytometry measurements of (*B*) PAC-1::FITC (*C*) CD62P::PE and (*D*) CD63::PECy5 induced by 6.25 μm hemin for 30 min at RT and preincubation with 1 μm PGE_1_ (5 min), 220 nm riociguat (5 min) + 0.5 μm DEA/NO (2 min) or the combination (PGE1 + riociguat + DEA/NO); plotted: Mean ± SD; n ≥ 3; statistics: Kruskal-Wallis test; Dunn`s multiple comparison (*B* and *C*). RM one-way ANOVA; Dunnett`s multiple Comparison (*D*). *E*, luminescence analysis of ATP-release induced with 6.25 μm hemin and if indicated preincubated with 1 μm PGE_1_ (5 min), 220 nm riociguat (5 min) + 0.5 μm DEA/NO or the triple combination at RT. plotted: Mean ± SD; n = 6; statistics: Friedman`s test; Dunn`s multiple comparison. *F*, heatmap with the percentual reduction of hemin-induced platelet aggregation achieved through administration of 1 μm PGE_1_ (5 min), 220 nm riociguat (5 min) + 0.5 μm DEA/NO (2 min) or the combination (PGE_1_ + riociguat + DEA/NO) at 37 °C. Darker shades of red indicate a stronger inhibitory effect.

Previously, it has been shown that elevated cGMP levels in platelets inhibit hemin-induced aggregation and activation (5). Therefore, we determined if modulation of the second messenger cAMP also inhibits hemin-induced platelet aggregation and whether simultaneous incubation of platelets with substances that increase cAMP (PGE_1_) and cGMP levels (riociguat + DEA/NO) exerts synergistic effects on platelet function. We found that platelet aggregation induced by low hemin concentrations was significantly inhibited by PGE_1_ (6.25 μm hemin *versus* 1 μm PGE_1_ + 6.25 μm hemin: *p* = 0.0015) (Fig. 1*A*). Simultaneous modulation of cGMP and cAMP levels also significantly inhibited the hemin-induced platelet aggregation, but not the cAMP effect alone (6.25 μm hemin *versus* PGE_1_ + riociguat + DEA/NO + 6.25 μm hemin: *p* = 0.0063) (Fig. 1*A*). In addition, activation of the fibrinogen receptor, α-degranulation, lysosomal degranulation and the ATP release from dense granules were significantly inhibited by PGE_1_ and the combination of PGE_1_ and riociguat + DEA/NO (Fig. 1, *B*–*E*). Interestingly, platelet inhibition was also observed when isolated human platelets were treated with high concentrations of hemin (12.5 and 25 μm) (Fig. 1*F*). Notably, simultaneous preincubation of platelets with PGE_1_ and riociguat + DEA/NO resulted in a synergistic inhibition of hemin-induced platelet aggregation at high concentrations of hemin (12.5 μm hemin *versus* PGE_1_ + riociguat + DEA/NO + 12.5 μm hemin: *p* < 0.001; 25 μm hemins *versus* PGE_1_ + riociguat + DEA/NO + 25 μm hemin: *p* < 0.05) (Fig. S1). The reduction in platelet aggregation is illustrated in the heatmap and highlights the synergistic effect of PGE_1_ combined with riociguat + DEA/NO (Fig. 1*F*). Darker shades of red indicate a stronger inhibitory effect (Fig. 1*F*). This suggests a synergistic interaction between two second-messenger pathways, particularly in platelet aggregation induced by high hemin concentrations.

### Synergistic effect of cAMP and cGMP prevents hemin-induced plasma membrane fragmentation

**Figure 2:**
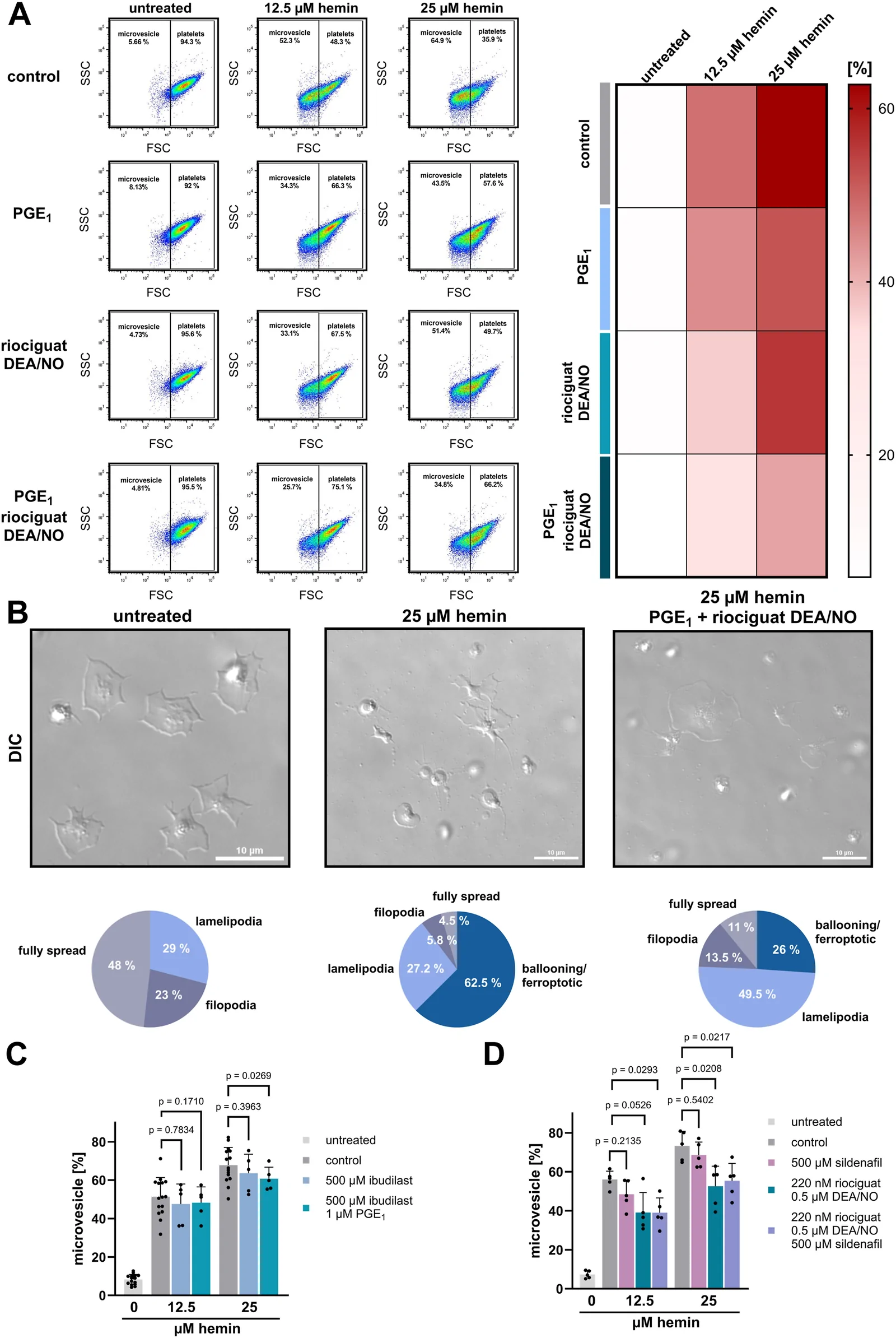
**Synergistic effect of cAMP and cGMP prevent hemin-induced plasma membrane fragmentation.***A*, flow cytometry measurements of microvesicles (<1 μm) after 30 min of incubation with 12.5 and 25 μm hemin and preincubation with 1 μm PGE_1_ (5 min), 220 nm riociguat (5 min) + 0.5 μm DEA/NO (2 min) or the combination (PGE_1_ + riociguat + DEA/NO) at RT. (*A*) *Left*: Representative dot plots showing the SSC/FSC and the threshold of microvesicle < 1 μm. *Right*: Heatmap showing the microvesicle formation [%] induced by 12.5 and 25 μm hemin and where indicated, preincubated with 1 μm PGE_1_ (5 min), 220 nm riociguat (5 min) + 0.5 μm DEA/NO (2 min) or the combination (PGE_1_ + riociguat + DEA/NO). Increasing intensity of the red signal corresponds to enhanced microvesicle formation. *B*, live-cell imaging of platelets on fibrinogen-coated coverslips (100 μg/ml); *upper row*: DIC images of untreated platelets, hemin-treated platelets or preincubated platelets with the combination of 1 μm PGE_1_ + 220 nm riociguat + 0.5 μm DEA/NO, *Lower raw*: Pie charts of platelet phenotype distribution; plotted: Mean in % from three different healthy donors; scale bar = 10 μm. *C*, flow cytometry measurements of microvesicle induced by 12.5 and 25 μm hemin and if indicated preincubated with 500 μm ibudilast (10 min) or the combination of 1 μm PGE_1_ + 500 μm ibudilast; plotted: Mean ± SD; n ≥ 5; statistics: Mixed-effects analysis; Dunnett`s multiple comparison. *D*, flow cytometry measurements of microvesicle induced by 12.5 and 25 μm hemin and if indicated preincubated with 500 μm sildenafil (10 min), 220 nm riociguat (5 min) + 0.5 μm DEA/NO (2 min) or the combination of sildenafil + riociguat + DEA/NO; plotted: Mean ± SD; n = 5; statistics: Mixed-effects analysis; Dunnett`s multiple comparison.

Recently, we have shown that especially high concentrations of hemin lead to plasma membrane disruption (3, 5, 9). Therefore, we wanted to determine whether modulating cAMP and cGMP levels affect this plasma membrane fragmentation. For this, isolated human platelets were preincubated with prostaglandin E_1_ (PGE_1_), riociguat + DEA/NO or all three (PGE_1_ + riociguat + DEA/NO) and subsequently challenged with 12.5 and 25 μm hemin. Microvesicle formation was measured to quantify the plasma membrane fragmentation (Fig. 2*A*). Surprisingly, the combination of PGE_1_ and riociguat + DEA/NO showed a pronounced inhibitory effect on microvesicle formation (12.5 μm hemin *versus* PGE_1_ + riociguat + DEA/NO: *p* < 0.001; 25 μm hemin *versus* PGE_1_ + riociguat + DEA/NO + 25 μm hemin: *p* < 0.001) (Figs. 2*A* and S2). In addition, hemin-induced phosphatidylserine (PS) exposure was inhibited (Fig. S3). The microvesicle formation and the inhibitory effects of PGE_1_ and riociguat + DEA/NO are presented in a heatmap, visualizing the synergistic effect of PGE_1_ and riociguat + DEA/NO (Fig. 2*A*). Increasing intensity of the red signal corresponds to enhanced microvesicle formation (Fig. 2*A*). Next, we examined if the combination of PGE_1_ and riociguat + DEA/NO influences the hemin-induced morphological changes in isolated human platelets. As previously described, hemin-treated platelets undergo morphological changes and lead to a ballooning/ferroptotic phenotype (3, 5, 9). Especially, the proportion of fully spread and filopodia-forming platelets was significantly reduced upon stimulation with 25 μm hemin, while the ballooning/ferroptotic phenotype was increased (fully spread: 48% in untreated *versus* 4.5% in 25 μm hemin; ballooning/ferroptotic: 0% in untreated *versus* 62.5% in 25 μm hemin) (Fig. 2*B*). Pre-treatment with PGE_1_ and riociguat + DEA/NO resulted in the reduction of the ballooning/ferroptotic phenotype (ballooning/ferroptotic: 62.5% in 25 μm hemin *versus* 26% in riociguat + DEA/NO + 25 μm hemin) (Fig. 2*B*). To investigate the effect of phosphodiesterase inhibition on the plasma membrane fragmentation, isolated human platelets were preincubated with ibudilast (PDE-3A inhibitor) and the combination of ibudilast and PGE_1_ (Fig. 2*C*). Interestingly, modulating the cAMP levels by ibudilast alone did not inhibit the hemin-induced microvesicle formation (Fig. 2*C*). Moreover, the combination of ibudilast and PGE_1_ did not exert a synergistic inhibitory effect (Fig. 2*C*). We further analyzed the impact of cGMP-modulating phosphodiesterase inhibitor sildenafil (PDE-5 inhibitor) on microvesicle formation (Fig. 2*D*). Sildenafil alone did not prevent microvesicle formation, nor did the combination with riociguat + DEA/NO result in a synergistic inhibitory effect (Fig. 2*D*). Notably, hemin-induced platelet aggregation was significantly reduced when platelets were preincubated with ibudilast (PDE-3 inhibitor) (*p* < 0.01) (Fig. S4). In contrast, sildenafil (PDE-5 inhibitor) does not significantly inhibit hemin-induced platelet aggregation (Fig. S4). Surprisingly, the combination of both inhibitors enhances the inhibitor effect (*p* < 0.001) (Fig. S4). These findings indicate that these two pathways are important for regulating hemin-induced platelet aggregation but are insufficient to prevent plasma membrane alterations. These results demonstrate that individual modulation of either the cGMP signaling pathway or the cAMP signaling pathway is insufficient to inhibit hemin-induced plasma membrane disintegration. In contrast, only the simultaneous targeting of both the cGMP and cAMP signaling pathways by modulatory compounds synergistically inhibits plasma membrane fragmentation.

### Hemin attenuates elevated cGMP levels induced by riociguat DEA/NO

**Figure 3:**
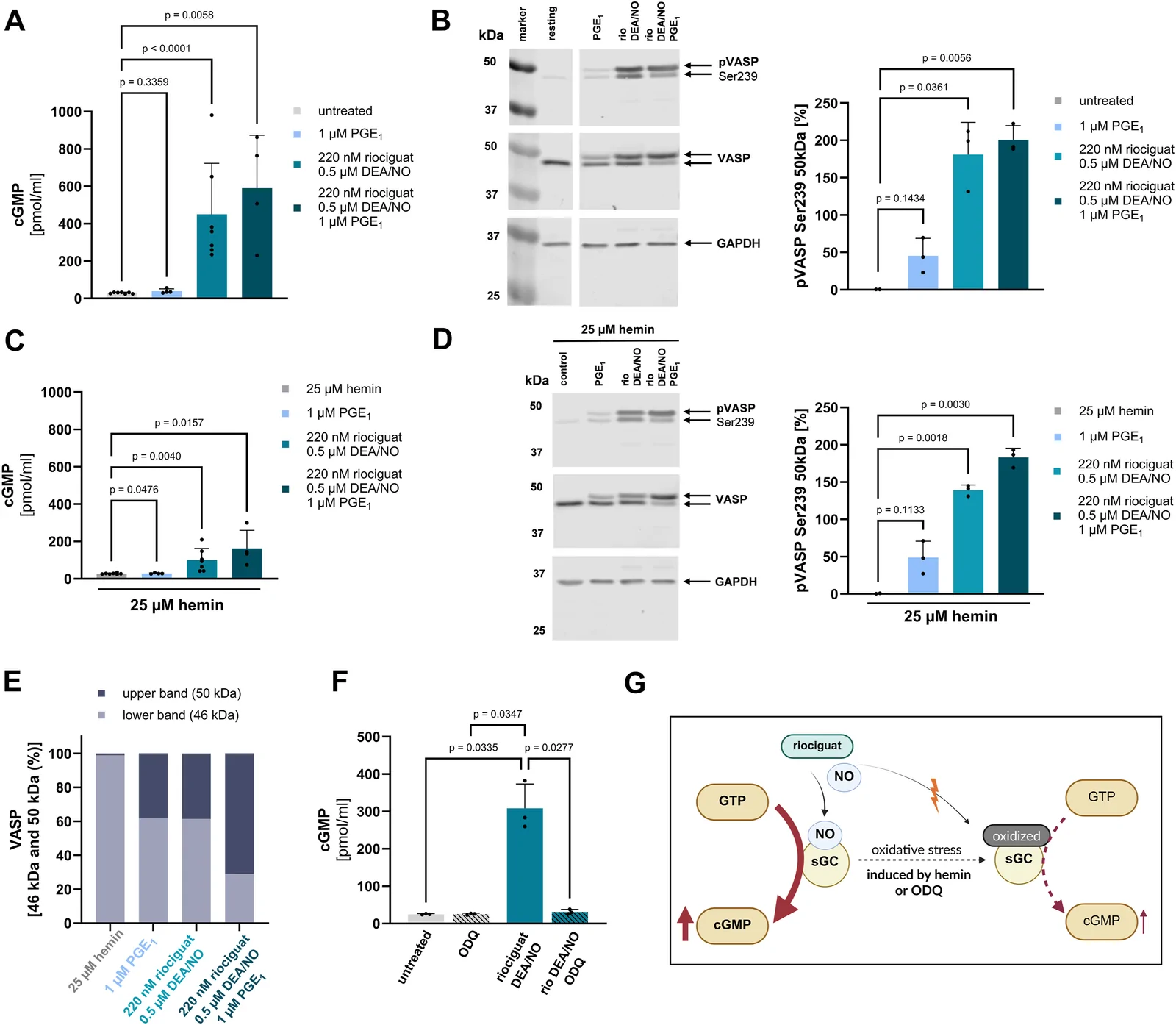
**Hemin attenuates elevated cGMP levels induced by riociguat DEA/NO.***A*, cGMP levels of isolated human platelets pretreated with 1 μm PGE_1_ (5 min), 220 nm riociguat (5 min) + 0.5 μm DEA/NO (2 min) or the combination (PGE_1_ + riociguat + DEA/NO) and incubated for 10 min at 37 °C; plotted: Mean ± SD; n ≥ 4; statistics: Mixed-effect analysis. *B*, *Left*: The representative immunoblot shows VASP phosphorylation at Ser239 under each condition. *Right*: Quantification of pVASP and statistical analysis of the pVASP (Ser239 at 50 kDa); plotted: Mean ± SD; n = 3; statistics: RM-one way ANOVA; Dunnett`s multiple comparison. *C*, cGMP levels of isolated human platelets pretreated with 1 μm PGE_1_ (5 min), 220 nm riociguat (5 min) + 0.5 μm DEA/NO (2 min) or the combination (PGE_1_ + riociguat + DEA/NO) and stimulated with 25 μm hemin for 10 min at 37 °C; plotted: Mean ± SD; n ≥ 4; statistics: Mixed-effect analysis. *D*, *Left*: The representative immunoblot shows VASP phosphorylation at Ser239 under each condition. *Right*: Quantification of pVASP and statistical analysis of the pVASP (Ser239 at 50 kDa); plotted: Mean ± SD; n = 3; statistics: RM-one way ANOVA; Dunnett`s multiple comparison. *E*, cumulative representation of the VASP distribution for each treatment. Plotted: Mean from three biological replicates. *F*, cGMP levels of isolated human platelets, where indicated pretreated with 10 μm ODQ (10 min) followed by incubation with 220 nm riociguat (5 min) + 0.5 μm DEA/NO (2 min) at 37 °C and if indicated stimulated with 25 μm hemin for 10 min at 37 °C; plotted: Mean ± SD; n = 3; statistics: RM-one way ANOVA; Dunnett`s multiple comparison. (*G*) Schematic illustration of the interaction between hemin and cGMP levels.

To further elucidate the molecular mechanisms underlying these findings, we measured the cGMP levels in platelets (Fig. 3*A*). Riociguat + DEA/NO significantly increased the cGMP levels, whereas PGE_1_ had no effect and the combination of PGE_1_ + riociguat + DEA/NO did not result in a synergistic increase in cGMP (untreated *versus* riociguat + DEA/NO: *p* < 0.0001) (Fig. 3*A*). Further, in the presence of riociguat + DEA/NO VASP Ser239 was significantly phosphorylated (untreated *versus* riociguat + DEA/NO: *p* = 0.036) (Fig. 3*B*). Uncropped blots are provided in the Supporting information (Fig. S5). Prostaglandin E_1_ alone induced a weaker phosphorylation of Ser239 (Fig. 3*B*). Interestingly, treatment of platelets with hemin reduced the riociguat + DEA/NO-induced cGMP synthesis, but still significantly increased the basal cGMP levels (25 μm hemin *versus* riociguat + DEA/NO + 25 μm hemin: *p* = 0.004) (Fig. 3*C*). Nevertheless, in the presence of hemin VASP phosphorylation at Ser239 was significantly increased (25 μm hemin *versus* riociguat + DEA/NO + 25 μm hemin: *p* = 0.0018) (Fig. 3*D*). Remarkably, the combination (PGE_1_ + riociguat DEA/NO) resulted in a strong shift of the VASP, as indicated by a mobility shift from 46 kDa to 50 kDa (Fig. 3*D*). Quantification of the band distribution of VASP showed that treatment with hemin alone resulted in nearly 100% localization of the protein in the lower band (46 kDa) (Figs. 3*E* and S6). In contrast, treatment with PGE_1_ or riociguat DEA/NO significantly shifted the distribution, with only approximately 60% remaining in the lower band (46 kDa) and about 40% appearing in the upper band (50 kDa). Importantly, the combined treatment with PGE_1_ and riociguat DEA/NO further significantly reduced the lower band (46 kDa) to approximately 30%, while increasing the upper band (50 kDa) to around 70% (Figs. 3*E* and S6). Therefore, the combined treatment results in a strong phosphorylation of VASP. The reduction of cGMP levels was also observed upon treatment with ODQ, a soluble guanylyl cyclase (sGC) inhibitor (riociguat + DEA/NO *versus* ODQ + riociguat + DEA/NO: *p* = 0.0277) (Fig. 3*F*). These findings suggest that hemin alters the oxidative state of the sGC in a manner similar to ODQ, thereby reducing riociguat + DEA/NO-mediated cGMP synthesis (Fig. 3*G*).

### cGMP-cAMP crosstalk and the phosphorylation of VASP at Ser157 and Ser239 are crucial for inhibition of hemin-induced plasma membrane disruption

**Figure 4:**
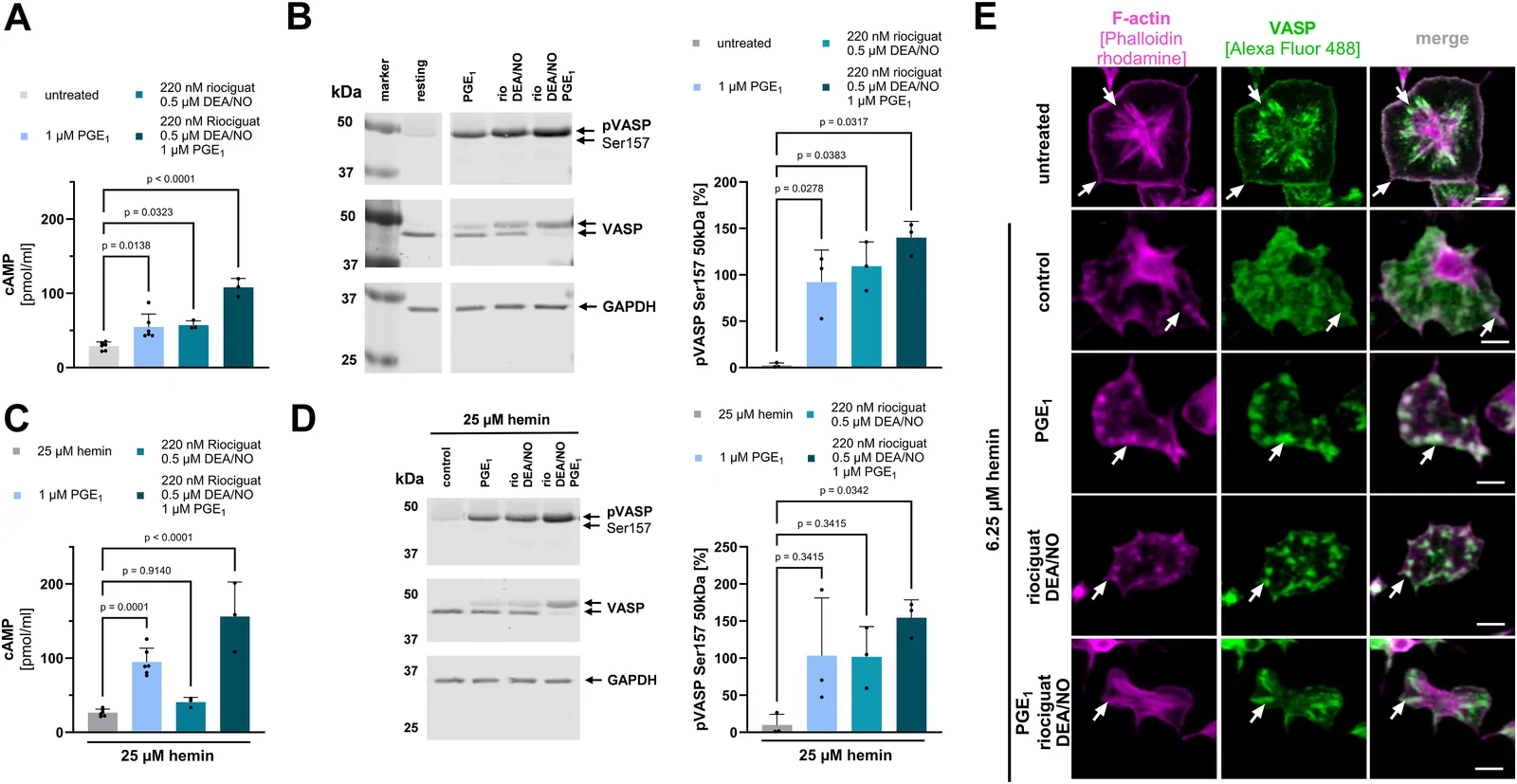
**cGMP-cAMP crosstalk and the phosphorylation of VASP at Ser157 and Ser239 are crucial for inhibition of hemin-induced plasma membrane disruption.***A*, cAMP levels of isolated human platelets pretreated with 1 μm PGE_1_ (5 min), 220 nm riociguat (5 min) + 0.5 μm DEA/NO (2 min) or the combination (PGE_1_ + riociguat + DEA/NO) and incubated for 10 min at RT; plotted: Mean ± SD; n ≥ 3; statistics: Two-way ANOVA; Tukey test. *B*, *Left*: The representative immunoblot shows VASP phosphorylation at Ser157 under each condition. *Right*: Quantification of pVASP and statistical analysis of the pVASP (Ser157 at 50 kDa); plotted: Mean ± SD; n = 3; statistics: RM-one way ANOVA; Dunnett`s multiple comparison. *C*, cAMP levels of isolated human platelets pretreated with 1 μm PGE_1_ (5 min), 220 nM riociguat (5 min) + 0.5 μm DEA/NO (2 min) or the combination (PGE_1_ + riociguat + DEA/NO) and stimulated with 25 μm hemin for 10 min at RT; plotted: Mean ± SD; n ≥ 3; statistics: Two-way ANOVA; Tukey test. *D*, *Left*: The representative immunoblot shows VASP phosphorylation at Ser157 under each condition. *Right*: Quantification of pVASP and statistical analysis of the pVASP (Ser157 at 50 kDa); plotted: Mean ± SD; n = 3; statistics: Friedmann`s test; Dunn`s multiple comparison. *E*, representative images of actin-staining (*magenta*) and immunofluorescence staining of VASP (*green*) in human platelets on fibrinogen coated-coverslips. Where indicated platelets were preincubated with 1 μm PGE_1_ (5 min), 220 nm riociguat (5 min) + 0.5 μm DEA/NO (2 min) or the combination (PGE_1_ + riociguat DEA/NO) at RT and stimulated with 6.25 μm hemin for 30 min at RT. White arrows showing F-actin and VASP localization. Scale bar = 1 μm.

Next, we measured the cAMP levels in platelets (Fig. 4, *A* and *C*). In contrast to cGMP, PGE_1_ significantly increased cAMP levels in platelets (untreated *versus* PGE_1_: *p* = 0.0138) (Fig. 4*A*). Remarkable, riociguat + DEA/NO also elevated cAMP levels to a similar extent as PGE_1_, and the triple combination of PGE_1_ + riociguat + DEA/NO resulted in a synergistic increase in cAMP levels (untreated *versus* PGE_1_ + riociguat DEA/NO: *p* < 0.0001) (Fig. 4*A*), indicating crosstalk between cGMP and cAMP signaling. Elevated cAMP levels are associated with increased phosphorylation of VASP at Ser157 (Fig. 4, *B* and *D*). Uncropped blots are provided in the Supporting information (Fig. S7). Unexpectedly, treatment of platelets with hemin in combination with PGE_1_ further increased cAMP levels beyond the effect of PGE_1_ alone (25 μm hemin *versus* PGE_1_ + 25 μm hemin: *p* = 0.0001) (Fig. 4*C*). Simultaneous treatment with riociguat + DEA/NO, PGE_1_ and the stimulation with 25 μm hemin resulted in an additional increase in cAMP levels (25 μm hemin *versus* PGE_1_ + riociguat DEA/NO + 25 μm hemin: *p* < 0.0001) (Fig. 4*C*). Furthermore, in the presence of 25 μm hemin, PGE_1_ strongly induced phosphorylation of VASP at Ser157 (Fig. 4*D*). Riociguat + DEA/NO likewise promoted phosphorylation at this site, further supporting the existence of crosstalk between cGMP and cAMP signaling pathway (Fig. 4*D*). Combined treatment resulted in significant synergistic phosphorylation of Ser157 and induced a complete electrophoretic shift of VASP (25 μm hemin *versus* PGE_1_ + riociguat DEA/NO + 25 μm hemin: *p* = 0.0342) (Fig. 4*D*). To investigate the localization of VASP and F-actin in the platelets we performed immunofluorescence of platelets on fibrinogen-coated coverslips (Fig. 4*E*). The IgG control is included in the Supporting information (Fig. S8). Untreated platelets exhibited full spreading and formed distinct F-actin filaments from the center of the platelet to the plasma membrane as well as a peripheral F-actin rim, which is co-localized to VASP (Fig. 4*E*). The resulting F-actin network of stress fibers are typical for spreading platelets. In contrast, hemin treatment markedly reduced the formation of F-actin filaments and thus network formation, resulting in a significant decrease in overall F-actin fluorescence intensity (Figs. 4*E* and S9). Pre-treatment with PGE_1_, riociguat DEA/NO and the combination (PGE_1_ + riociguat DEA/NO) significantly increased the mean fluorescence F-actin signal compared to hemin-treated platelets, indicating modulation of cAMP- and cGMP-depending signaling attenuated the hemin-induced loss of F-actin (Fig. S9). But both compounds induce the formation of nodules-like structures of F-actin not fibers, which results in punctual accumulation of F-actin and therefore overall stronger F-actin fluorescence intensity (Figs. 4*E* and S9). In contrast, total-VASP signal intensity slightly increased in hemin-treated platelets, because it did not continue to co-localize with the remaining F-actin structures, which were less pronounced formed (Figs. 4 and S9). Notably, in hemin-treated platelets, total-VASP was also detected in the cytosol, independent of F-actin fibers, showing remaining total-VASP upon hemin exposure (Fig. 4*E*). Interestingly, the combination of PGE_1_ and riociguat/DEA-NO resulted in F-actin staining patterns that correspond to the F-actin network observed in untreated platelets (Figs. 4*E* and S9). The F-actin signal was significantly increased, suggesting that the treatment significantly attenuated hemin-induced F-actin depolymerization and preserved the ability of platelets to form long F-actin filaments and the resulting F-actin networks. The intensity of the VASP signal in PGE_1_ + riociguat DEA/NO-treated platelets was similar to that of untreated platelets (Fig. S9). However, the intracellular distribution of VASP appeared to be altered. In contrast to untreated platelets, VASP was less co-localized with F-actin filaments and closer to the plasma membrane (Fig. 4*E*). We therefore speculate that this contributes to the pronounced stabilization of the plasma membrane, thereby preventing hemin-induced plasma membrane fragmentation.

## Discussion

The main findings of this study are (1) Modulation of cGMP and cAMP synergistically inhibits platelet aggregation and plasma membrane fragmentation induced by high concentrations of hemin (25 μm) (2). cGMP-cAMP crosstalk and the phosphorylation of VASP at Ser157 and Ser239 are crucial for inhibition of the hemin-induced plasma membrane disruption (3). Hemin changes the oxidative state of the soluble GC (4). Hemin and PGE_1_ synergistically increase cAMP levels in platelets.

Only recently, we demonstrated that hemin induces platelet activation through both GPVI- and CLEC-2-dependent mechanisms in a concentration-dependent manner (3). Accordingly, both low and high concentrations of hemin promote platelet activation, degranulation, and aggregation. However, important differences emerge at higher hemin concentrations. While platelet activation induced by lower concentrations of hemin is largely mediated through GPVI and CLEC-2 signaling, higher concentrations additionally trigger receptor-independent mechanisms (3). In particular, microvesicle formation and plasma membrane disruption are no longer fully prevented by blockade of GPVI and CLEC-2, indicating the activation of alternative pathways that bypass classical receptor signaling (3). Therefore, the differences between low- and high-dose hemin responses are unlikely to be explained solely by distinct signaling through monomeric *versus* dimeric CLEC-2. Rather, high concentrations of hemin appear to circumvent receptor-dependent pathways altogether. This is consistent with the ability of hemin to directly intercalate into lipid membranes. At lower concentrations, hemin predominantly interacts with platelet surface receptors such as GPVI and CLEC-2, whereas at higher concentrations it additionally interferes with the plasma membrane itself, resulting in membrane damage, microvesicle release, and receptor-independent platelet activation.

In this study, we further wanted to find potential pharmacological influencing factors to inhibit the receptor-independent signaling pathway. The focus was to evaluate the receptor-independent pathway by applying cAMP and cGMP modulating agents to platelets treated with high concentrations of hemin. We found that the concentration-dependent hemin-induced platelet aggregation was inhibited by elevating cAMP and cGMP levels. Interestingly, inhibition of the platelet aggregation induced by high concentrations of hemin was potentiated if both cAMP- and cGMP-modulating substances were applied. In comparison to conventional anti-platelet therapy with cangrelor and indometacin, cAMP and cGMP modulating drugs inhibit platelet aggregation induced by both low and high concentrations hemin. This indicates that the cAMP and cGMP pathway contribute to the inhibition of the receptor-independent pathway, suggesting a stabilization of the plasma membrane. Indeed, we also measured a synergistic effect of cAMP and cGMP-modulating substances, when analyzing the microvesicle formation as well as the ballooning/ferroptotic morphological changes under high concentrations of hemin. Notably, inhibition of the second messenger degradation by ibudilast (PDE3 inhibitor) and in combination with PGE_1_, as well as sildenafil (PDE5 inhibitor) in combination with riociguat + DEA/NO, did not potentiate the inhibitory effect on microvesicle formation (23). Nevertheless, these compounds effectively inhibited hemin-induced platelet aggregation, suggesting that effective inhibition of hemin-induced membrane changes requires activation of both major inhibitory pathways. Ibudilast exerts as PDE-3 inhibitor anti-platelet effects by reducing platelet aggregation (23). Furthermore, ibudilast inhibits MIF signaling, thereby decreasing the release and activity of platelet-derived MIF (24). Platelet-derived MIF is a key mediator of thromboinflammation because it enhances interactions between platelets and monocytes/macrophages, thereby amplifying platelet activation and inflammatory responses (25, 26, 27). We speculate that hemin induces the release of platelet-derived macrophage migration inhibitory factor (MIF), which can be inhibited by ibudilast. As a result, platelet degranulation, thromboinflammation, and platelet–monocyte/macrophage interactions are attenuated. This reduces the inflammatory potential of activated platelets and may limit platelet-dependent thrombosis as well as the inflammatory processes through hemin.

Previously, we already demonstrated that modulating the NO/cGMP/PKG pathway inhibits hemin-induced platelet function and lipid peroxidation (5, 13, 28). The NO/cGMP/PKG and the cAMP/PKA pathway are the major inhibitory pathways in platelets and can be modulated by several pharmacological substances (13, 28). cGMP is synthesized by the NO-sensitive soluble guanylyl cyclase. sGC can be inactivated by oxidation, which leads to the loss of the NO-binding heme group (17). The oxidative inactivation can occur under conditions of increased oxidative stress or upon treatment with the sGC inhibitor, ODQ (29). As hemin is known to induce oxidative stress, we postulate that hemin alters the redox state of sGC, similar to ODQ (10, 30). Therefore, impairing NO-binding and reducing riociguat DEA/NO-induced cGMP synthesis. Intracellular cGMP and cAMP levels are regulated by phosphodiesterases (PDEs), which degrade the second messengers (22). Further, cGMP inhibits PDE3, thereby reducing cAMP degradation. In our study, we observed this cGMP-cAMP crosstalk. In particular, in the presence of high concentrations of hemin, the combination of cGMP and cAMP-modulating drugs, increased the cAMP levels.

Our data indicate that simultaneous targeting of both the cAMP and cGMP pathway is required to potentiate hemin-induced plasma membrane fragmentation. This effect is accompanied by a shift of the VASP protein from 46 to 50 kDa. The shift represents the hyperphosphorylation state of VASP were Ser157 and Ser239 are both phosphorylated. VASP is a key regulator of the cytoskeleton and is localized to F-actin-rich structures. It modulates actin remodeling by promotion profilin recruitment, bundling and filament formation and the anti-capping and anti-branching of the F-actin (31, 32, 33). In resting platelets 40% of total actin is present in F-actin filaments, increasing to about 80% upon activation. Phosphorylation of VASP reduces the affinity for actin and is associated with decreased F-actin polymerization. VASP gets phosphorylated from cGMP and cAMP (34, 35). Treatment with PGE_1_ and riociguat + DEA/NO inhibits F-actin polymerization and induced the formation of F-actin nodules, which are localized on the plasma membrane, similar to the nodules formed during early platelet spreading. These structures appear to stabilize the membrane through a connecting bridge of talin and vinculin between the nodules and the membrane. This could prevent uncontrolled blebbing of the membrane, thereby reducing microvesicle release. Notably, PGE1 + riociguat + DEA/NO synergistically increased the cAMP levels, but not the cGMP levels in platelets, suggesting cGMP-cAMP crosstalk mediated through the inhibition of PDE3. The combination of PGE_1_ and hemin also potentiated the cAMP accumulation in platelets, suggesting a protective negative-feedback mechanism. Raslan et *al*. demonstrated that the disruption of lipid rafts using methyl-beta-cyclodextrin (MβCD)-enhanced platelet sensitivity to PGI_2_ and forskolin, a direct AC cyclase activator, resulting in greater inhibition of collagen-stimulated platelet aggregation (36). It is therefore that hemin disrupts the lipid raft integrity and breaks down cAMP compartmentalization. This may result in amplified cAMP signaling, to prevent hemin-induced platelet activation and plasma membrane fragmentation.

In summary, cGMP-and cAMP- modulating drugs elevate intracellular cGMP and cAMP levels in human platelets stimulated with 25 μm hemin, leading to synergistic phosphorylation of VASP at Ser239 and Ser157, which results in a complete shift of the VASP protein to the hyperphosphorylated state (50 kDa). These molecular effects are crucial for the inhibition of hemin-induced plasma membrane fragmentation and prevent microvesicle formation.

The simultaneous administration of cAMP- and cGMP-modulating drugs may lead to hypotensive crisis and could therefore be counterproductive in combination. This study is primarily intended to provide a deeper molecular understanding of hemin and its potential interacting pathway in platelets. From this, it can be concluded that targeting cGMP or cAMP pathway, not only hemin-induced platelet activation and aggregation but also hemin-induced plasma membrane fragmentation could be prevented.

## Experimental procedures

### Chemicals and antibodies

Hemin (cat. no. H9039), fibrinogen from human plasma (cat. no. F3879), prostaglandin E_1_ (PGE_1_, catalog number P5515) was obtained from Sigma-Aldrich. Ibudilast (cat. no. HY-B0763) and sildenafil (cat. no. HY-15025) was purchased from MedChemExpress (Monmouth Junction). Riociguat was ordered from AMbeed (Buffalo Grove; cat. no. A255332). Diethylamine nonoate (DEA/NO; cat. no. ab145197) was obtained from Abcam. ODQ was obtained from Tocris (Bristol, UK; cat. no. 0880). IBMX (3-Isobutyl-1-methyl-Xanthin; cat. no. I5879) and DMSO (cat. no. D8418) was purchased by Merck. Protease cocktail (cat. no. 5871) and protease/phosphatase inhibitor (cat. no. 5872) were obtained from Cell Signaling Technology. SDS containing loading buffer was ordered from Carl Roth GmbH + Co. KG (Karlsruhe, Germany cat. no 1057). PVDF membrane was purchased from Sigma-Aldrich (cat. no. IPFL00010). Bovine serum (BSA) was obtained from Applichem (cat.no. 1198523).

Monoclonal anti-human PAC-1::FITC antibody was obtained from BD Bioscience (clone PAC-1; catalog number 340507). Anti-human CD62P::PE antibody were obtained from Beckman Coulter (Clone CLB-Thromb/6; catalog number IM1759U). Anti-human CD63:: PE/Cy5 antibody (Clone MEM-259; catalog number ab234251) was obtained from Abcam. A flow cytometer size calibration kit was ordered from Invitrogen (cat. no F13838).

Anti-VASP (clone: 9A2; cat. no. 3132), anti-phospho-VASP (Ser239; clone: E9L7Y; cat. no. 37565), anti-phospho-VASP (Ser157; clone: D1C80; cat. no. 84519), and rabbit monoclonal antibody IgG control (clone: DA1E; cat. no. 3900) were all obtained from Cell Signaling Technology. Anti-GAPDH antibody (clone: GA1R; D1080; cat. no. MA5-15738) was obtained from Invitrogen. Donkey anti-rabbit IR-Dye 680RD (cat. no. 925-68073), donkey anti-rabbit IR-Dye 800CW (cat. no. 925-32213) and donkey anti-mouse IR-Dye 800CW (cat. no. 925-32212) secondary antibodies were obtained from Li-COR (Lincoln, NE, USA). Phalloidin labeling probe (Rhodamine phalloidin; cat. no. R415) and the secondary antibody AlexaFlour 488 (cat. no. A48282TR) were obtained from Invitrogen.

### Isolation of human platelets

Blood samples were collected from healthy donors who provided written informed consent. The procedure was approved by the Ethics Committee of the Medical Faculty of the Eberhard Karls University and the University Hospital of Tübingen (ethics vote 238/2018B02) and was conducted in accordance with the principles of the Declaration of Helsinki. Platelet isolation was performed as previously described (3, 37, 38). Blood was drawn into syringes containing acid-citrate-dextrose (ACD) anticoagulant. To obtain platelet-rich plasma (PRP), samples were centrifuged at 209*g* for 20 min without brakes. The PRP was carefully transferred into Tyrode’s buffer (pH 6.5) (137 mm NaCl, 2.8 mm KCl, 12 mm NaHCO_3_, 5 mm glucose, 10 mm HEPES). Then, the PRP was centrifuged at 836*g* for 10 min with brakes applied. The supernatant was discarded, and the platelet pellet was resuspended in Tyrode’s buffer (pH 7.4). Platelet concentration was determined using a conventional cell counter (Sysmex Coorporation).

### Flow cytometry

Isolated human platelets were adjusted to a count of 1 × 10^6^ per sample in Dulbecco’s phosphate buffered saline (0.5 mm MgCl_2,_ 0.9 mm CaCl_2_). First, the platelets were preincubated with 1 μm PGE1 (5 min), 220 nm riociguat (5 min) + 0.5 μm DEA/NO (2 min) or the triple combination of PGE_1_ + riociguat + DEA/NO. Then, samples were stimulated at indicated concentrations with hemin for 30 min at room temperature in the presence of PAC-1::FITC (1:20), CD62P::PE (1:20) or CD63::PECy5 (1:25) monoclonal antibodies. The reaction was stopped by the addition of 250 μl 0.5% paraformaldehyde solution (PFA). Measurements were performed using a FACS Calibur (BD Biosciences) and FACSLyric (BD Bioscience).

Microvesicle formation was also measured by flow cytometry, as previously described (3, 5, 37). Isolated human platelets were prepared as described above. Platelets were preincubated min 1 μm PGE_1_ (5 min), 220 nm riociguat (5 min) + 0.5 μm DEA/NO (2 min) or the combination and 500 μm sildenafil (10 min) or 500 μm ibudilast (10 min) and stimulated with hemin for 30 min at room temperature. Then, the samples were fixed with 0.5% paraformaldehyde, and data were collected to 30,000 events using FSC/SSC signals by FACSLyric (BD Bioscience). To define the microvesicle gate, a suspension of 1 μm beads from a flow cytometer size calibration kit was analyzed, allowing identification of cells smaller than 1 μm, which are by definition the microvesicles. The percentage of microvesicles was calculated as the proportion of events <1 μm relative to the total platelet event count. The raw data of all flow cytometer measurements were analyzed using FlowJo software (version 10.10.0 FlowJo CCL).

### Light transmission aggregometry

Light transmission aggregometry experiments were performed with washed platelets adjusted to 2 × 10^7^ per sample, as previously described (5, 39). Where indicated, platelets were preincubated with 1 μm PGE_1_ (5 min), 220 nM riociguat (5 min) + 0.5 μm DEA/NO (2 min), or the combination (PGE_1_ + riociguat + DEA/NO) at 37 °C. Immediately after that, 2 mm Ca^2+^ and 100 μg/ml fibrinogen were added to the platelets, and the aggregation was induced by hemin and measured using a light transmission aggregometer (CHRONO-LOG Aggregometer 490, Chrono-log Corporation) over 5.30 min at 37° with continuous stirring at 1000 rpm.

### ATP-release

ATP-release was measured as previously described (39). In short, to measure ATP-release, isolated human platelets were adjusted to a concentration of 200 × 10^6^ platelets per μl. Platelet suspension was then pretreated with PGE_1_ (1 μm) for 5 min at 37 °C, 220 nm riociguat (5 min) + 0.5 μm DEA/NO (2 min), combination or respective control. Next, calcium (2 mm) and fibrinogen (100 μg/ml) were added before agonist treatment with 6.25 μm hemin. After calibration of one sample with ATP standard (ChronoLog), the ATP concentration was determined utilizing the ChronoLume luciferin assay (ChronoLog) on a luminoaggregometer (Modell 700, ChronoLog) according to the manufacturer's protocol.

### Immunoblot analysis

VASP signaling was analyzed as previously described (5, 23). Where indicated, isolated human platelets (2.5 × 10^8^ per sample) were preincubated with 1 μm PGE_1_ (5 min), 220 nm riociguat (5 min) + 0.5 μm DEA/NO (2 min) at room temperature (RT) prior stimulation with hemin for 10 min at RT. After that, cells were lysed with RIPA lysis buffer (50 mm TRIS/HCl, 150 mm NaCl, 0.1% SDS, 1% Triton-X 100, 0.5% Na-Deoxycholate) supplemented with protease cocktail (1:100) and protease/phosphatase inhibitor (1:100) and incubated for at least 10 min on ice. Platelet lysates were mixed with sodium dodecyl sulfate (SDS) containing loading buffer and separated by sodium dodecyl sulfate-polyacrylamide gel electrophoresis (SDS-PAGE) using a 10% polyacrylamide gel. Proteins were transferred to a PVDF membrane using a wet blot technique for 65 min at 100 V. Membranes were blocked for 1 h at room temperature with 5% bovine serum (BSA) in Tween-Tris buffered saline (TTBS), followed by overnight incubation at 4 °C with the primary antibodies (1:500 anti-phospho-VASP). The next day, the membranes were labeled with appropriate IRDye-conjugated secondary antibodies (1:15,000) for 90 min at room temperature. Equal protein loading was confirmed by anti-GAPDH staining (1:2000). After drying, membranes were scanned and analyzed by Li-COR- Odyssey System (LI-COR Biotechnology GmbH). The antibody signal was validated by the molecular weight pattern visible on the scanned blot, which concurs with the molecular weight of VASP/pVASP. Both the VASP and the pVASP antibody align at the same molecular weight, further validating the observed signal.

### Live-cell imaging on fibrinogen

Fibrinogen-coated coverslips (100 μg/ml) were incubated over night at 4 °C. The following day, the coverslips were washed with Dulbecco`s phosphate buffered solution (PBS) and blocked with 1% bovine serum (BSA) for 1 h at room temperature. Isolated platelets were adjusted to 20,000 platelets/μl with 1 mm CaCl_2_. Where indicated platelets were preincubated with the combination of 1 μm PGE_1_ (5 min) + 220 nm riociguat (5 min) + 0.5 μm DEA/NO (2 min) at RT and treated with 25 μm hemin. After 30 min of stimulation, images were taken using a Nikon Eclipse Ti2 microscope (Nikon Instruments Europe BV). For each treatment condition, five randomly selected areas were analyzed from three independent healthy donors, and morphological phenotypes were quantified by counting the respective cell types.

### Immunofluorescence of VASP and staining of F-actin

For VASP and F-actin staining, isolated human platelets were prepared as described for live-cell imaging. Following preincubation with 1 μm PGE_1_ (5 min), 220 nm riociguat (5 min) + 0.5 μm DEA/NO (2 min), platelets were stimulated with hemin for 30 min at RT. Samples were fixed with 2% paraformaldehyde for 15 min. Permeabilization was performed using 0.1% Trition-X-100 in PBS for 5 min. After three washing steps, samples were blocked with 1% BSA for 1 h at RT. Platelets were incubated overnight at 4 °C with the primary antibody (anti-VASP; 9A2; 1:400) or a corresponding IgG control. The following day, samples were incubated with the secondary antibody (Alexa Fluor anti-rabbit; 1:500) for 30 min at RT. F-actin was stained by incubation with Phalloidin rhodamine (1:200) for 1h at RT. Finally, the coverslips were mounted onto slides for imaging. Images were taken with a confocal laser-scanning-microscope (Leica Stellaris 5, Leica, LMB R039a) and processed with the software LasX (Leica). Images of the IgG controls were taken at the same settings as the anti-VASP-stained samples, to validate the anti-VASP signal.

### Measurement of cGMP and cAMP levels

To determine the levels of cyclic GMP (cat. no. ADI-900-031) and cyclic AMP (cat. no. ADI-900-067A) in human platelets an enzyme-linked immunosorbent assay (ELISA, Enzo Life Sciences) was used as previously described (40). Isolated human platelets were adjusted to 1.5 × 10^6^ per μl. If indicated, platelets were preincubated with 1 μm PGE_1_ (5 min), 220 nm riociguat (5 min) + 0.5 μm DEA/NO (2 min) or the combination (PGE_1_ + riociguat + DEA/NO). Then, the platelets were treated for 10 min with hemin at 37 °C. After that, samples were lysed with a lysis buffer containing 0.5% Triton-X-100 and 1 mm IBMX to stop endogenous phosphodiesterase activity. After centrifugation at 12,000 rpm for 5 min the supernatant was used to perform the ELISA according to the manufacturer`s instructions.

### Statistics and graphical presentation

Statistical analysis and graphical data presentation were performed using GraphPad Prism (Version 10.1.1; GraphPad software, Inc., Version 10.1.1). All datasets were tested for normal distribution, using GraphPad Prism the normality and lognormality tests. For more than two comparisons of normal distributed data, an RM one-way ANOVA, two-way ANOVA or mixed-effects analysis were used. For not normal distributed data, a Friedman's test or the Kruskal-Wallis test were performed. Data are presented as mean ± standard deviation (SD), with individual data points super-imposed. The data points depict biological replicates, if not otherwise stated, in the figure legends. Statistical significance was defined as *p* < 0.05. The graphical abstract and further illustrations were created with BioRender.

## Data availability

For original data please contact meinrad.gawaz@med.uni-tuebingen.de.

## Supporting information

This article contains supporting information.

## Conflict of interest

The authors declare that they have no conflicts of interest with the contents of this article.
