# Supplementary material for: Synergistic modulation of cAMP and cGMP rescues hemin-induced plasma membrane fragmentation
Source: J Biol Chem. 2026 Jul 24;302(9):113357. doi: 10.1016/j.jbc.2026.113357 (PMC13503134; doi:10.1016/j.jbc.2026.113357)
Supplement: Supplementary file 1 [file mmc1.docx]

# **Supporting information**

**Synergistic modulation of cAMP and cGMP rescues hemin-induced plasma membrane fragmentation**

Zoi Laspa^1*^, Anne-Katrin Rohlfing^1*^, Melina Fischer^1,2^, Ferdinand Kollotzek^1,2^, Tatsiana Castor^1^, Oliver Borst^1,2^, Pamela Weronika Sowa^1^, Manuel Sigle^1^, Tobias Harm^1^, Meinrad Paul Gawaz^1^

*Contributed equally

^1^Department of Cardiology and Angiology, University Hospital Tübingen, University Tübingen, Tübingen, Germany.

^2^DFG Heisenberg Group Cardiovascular Thrombo-Inflammation and Translational Thrombocardiology, University of Tübingen, Tübingen, Germany.

**Corresponding author**

Meinrad P. Gawaz, MD

Department of Cardiology and Angiology; University Hospital Tübingen; Eberhard Karls University Tübingen

Otfried-Müller-Str. 10; 72076 Tübingen, Germany

Tel.: +49 7071 29 83688; Fax: +49 7071 29 5749

E-Mail: meinrad.gawaz@med.uni-tuebingen.de

**Figure S1**

**Supporting Figure S1. Light transmission aggregometry measurements.** Left: Representative traces of **(A)** 12.5 and **(B)** 25 µM hemin-induced aggregation and if indicated preincubated with 1 µM PGE_1_ (5 min), 220 nM riociguat (5 min) + 0.5 µM DEA/NO (2 min) or the combination (PGE1 riociguat DEA/NO) at 37°C. Right: Hemin-induced maximal aggregation after 5 min at 37°C and preincubation with 1 µM PGE1 (5 min), 220 nM riociguat (5 min) + 0.5 µM DEA/NO (2 min) or the combination (PGE_1_ + riociguat + DEA/NO); plotted: Mean ± SD; n ≥ 3; statistics: Mixed-effects analysis, * p < 0.05, **p < 0.01, ***p < 0.001.

**Figure S2**

**Supporting Figure S2. Flow cytometry measurement of microvesicles** (< 1µm) after 30 min of incubation with 12.5 and 25 µM hemin and preincubation with 1 µM PGE_1_ (5 min), 220 nM riociguat (5 min) + 0.5 µM DEA/NO (2 min) or the combination (PGE_1_ + riociguat + DEA/NO) at RT; plotted: Mean ± SD; n ≥ 7; statistics: Mixed-effects analysis, * p < 0.05, ***p < 0.001.

**Figure S3**

**Supporting Figure S3: Flow cytometry measurements of hemin-induced phosphatidylserine exposure.** Isolated human platelets were activated with 12.5 and 25 µM hemin after pre-incubation with 1 µM PGE_1_ (5 min), 220 nM riociguat (5 min) + 0.5 µM DEA/NO (2 min) or the combination (PGE_1_ + riociguat + DEA/NO) for 30 min at RT. Plotted: Mean ± SD; n = 4; statistics: Friedman test; Dunn`s multiple comparison test. * p < 0.05.

**Figure S4**

**Supporting Figure S4:** Effect of phosphodiesterase inhibitors on hemin-induced platelet aggregation. Isolated human platelets were activated with 25 µM hemin after pre-incubation with **A** 500 µM ibudilast (10 min) at 37°C. Left: Representative traces. Right: maximal aggregation after 5.30 min. Plotted: Mean ± SD; n = 5; statistics: paired t test; ** p < 0.01. **B** 500 µM sildenafil (10 min) at 37°C. Left: Representative traces. Right: Plotted: Mean ± SD; n = 3; statistics: Wilcoxon matched-pairs signed rank test. And **C** Combination of ibudilast and sildenafil at 37°C. Left: Representative traces. Right: Plotted: Mean ± SD; n = 4; statistics: RM one-way ANOVA, Dunnet`s multiple comparisons test. *** p<0.001.

**Figure S5**

**Supporting Figure S5: Full images of representative western blot.** Upper row: Illustrating VASP phosphorylation at Ser239. Middle row: Illustrating VASP. Lower row: Illustrating the loading control GAPDH. Samples were preincubated with 1 µM PGE_1_ (5 min), 220 nM riociguat (5 min) + 0.5 µM DEA/NO (2 min) or the combination (PGE_1_ + riociguat + DEA/NO) and if indicated activated with 25 µM hemin for 10 min at 37°C.

**Figure S6**

**Supporting Figure S6: Quantification of VASP after immunoblot analysis.** Isolated human platelets were incubated with 1 µM PGE_1_, 220 nM riociguat + 0.5 µM DEA/NO or the combination (PGE1 + riociguat + DEA/NO) prior treatment with 25 µM hemin. **A** Percentage-based analysis of the lower VASP band (46 kDa). Plotted: Mean ± SD; n = 3; statistics: RM one-way ANOVA, Dunnet`s multiple comparisons test. * p < 0.05, ** p < 0.01. **B** Percentage-based analysis of the upper VASP band (50 kDa). Plotted: Mean ± SD; n = 3; statistics: RM one-way ANOVA, Dunnet`s multiple comparisons test. * p < 0.05, ** p < 0.01.

**Figure S7**

**Supporting Figure S7: Full images of representative western blot.** Upper row: Illustrating VASP phosphorylation at Ser157. Middle row: Illustrating VASP. Lower row: Illustrating the loading control GAPDH. Samples were preincubated with 1 µM PGE_1_ (5 min), 220 nM riociguat (5 min) + 0.5 µM DEA/NO (2 min) or the combination (PGE_1_ + riociguat + DEA/NO) and if indicated activated with 25 µM hemin for 10 min at 37°C.

**Figure S8**

**Supporting Figure S8: Representative immunofluorescence images of actin-staining (magenta) and IgG control staining of VASP (green) in human platelets on fibrinogen coated coverslips.** Platelets were not treated. Upper scale bar = 3 µm. Lower scale bar = 1 µm.

**Figure S9**

**Supporting Figure S9: Quantification of the immunostaining Analysis.** Measurement of single platelets from ≥ 3 different healthy donors; plotted: Mean of ≥ 100 single platelets per condition. If indicated, preincubated with 1 µM PGE_1_, 220 nM riociguat + 0.5 µM DEA/NO and the combination (PGE_1_ + riociguat + DEA/NO) and activated with 6.25 µM hemin for 30 min at RT. **A Mean** **fluorescence intensity (MFI) of rhodamine (F-actin).** Statistics: Ordinary one-way ANOVA, ***p < 0.001, ****p < 0.0001. **B Mean fluorescence intensity (MFI) of Alexa Fluor 488 (Total-VASP).** Statistics: Kruskal-Wallis test, ** p < 0.01, ****p < 0.0001.
